# Adult Small Bowel Intussusception and Resolution Captured on Pelvic Ultrasound

**DOI:** 10.7759/cureus.5622

**Published:** 2019-09-11

**Authors:** Neil P Larson, Michael J Yoo, Rachel E Bridwell

**Affiliations:** 1 Emergency Medicine, Brooke Army Medical Center, Fort Sam Houston, USA

**Keywords:** adult intussusception, abdominal pain

## Abstract

While abdominal pain is one of the leading causes for ED visits, intestinal intussusception is an infrequent etiology in adults. We present the case of a 22-year-old woman with five days of left lower quadrant abdominal pain with initial workup investigating for a genitourinary source utilizing pelvic ultrasound which revealed small bowel-small bowel intussusception. During the study, resolution of the intussusception correlated temporally with the patient’s symptoms. Transient small bowel intussusception in adults has been previously described; however, direct visualization of both the intussusception and its resolution on pelvic ultrasound has not been previously published.

## Introduction

Abdominal pain is one of the most common chief complaints seen in the ED, accounting for approximately 5% of all chief complaints [1]. In a young woman of childbearing age, this differential diagnosis is extremely broad. Intussusception, or telescoping of one bowel segment into another, is an infrequent etiology of abdominal pain in adults, with only 5% of all intussusceptions diagnosed in the adult population [2]. While the majority of large bowel intussusception lead points are malignant in nature, those involving the small bowel are often more benign [3]. Though malignant lesions still compose up to 30% of small bowel intussusception lead points, other recognized sources include lipomas, adenomas, and neurofibromas among others [2, 4]. However, up to 20% of adult intussusceptions are idiopathic without identified lead points, with higher incidence occurring in the small bowel [2]. Transient, nonobstructing small bowel-small bowel intussusceptions may also be seen in symptomatic patients during diagnostic imaging, and are more likely idiopathic in nature without long-term clinical sequelae [3-6].

## Case presentation

**Figure 1:**
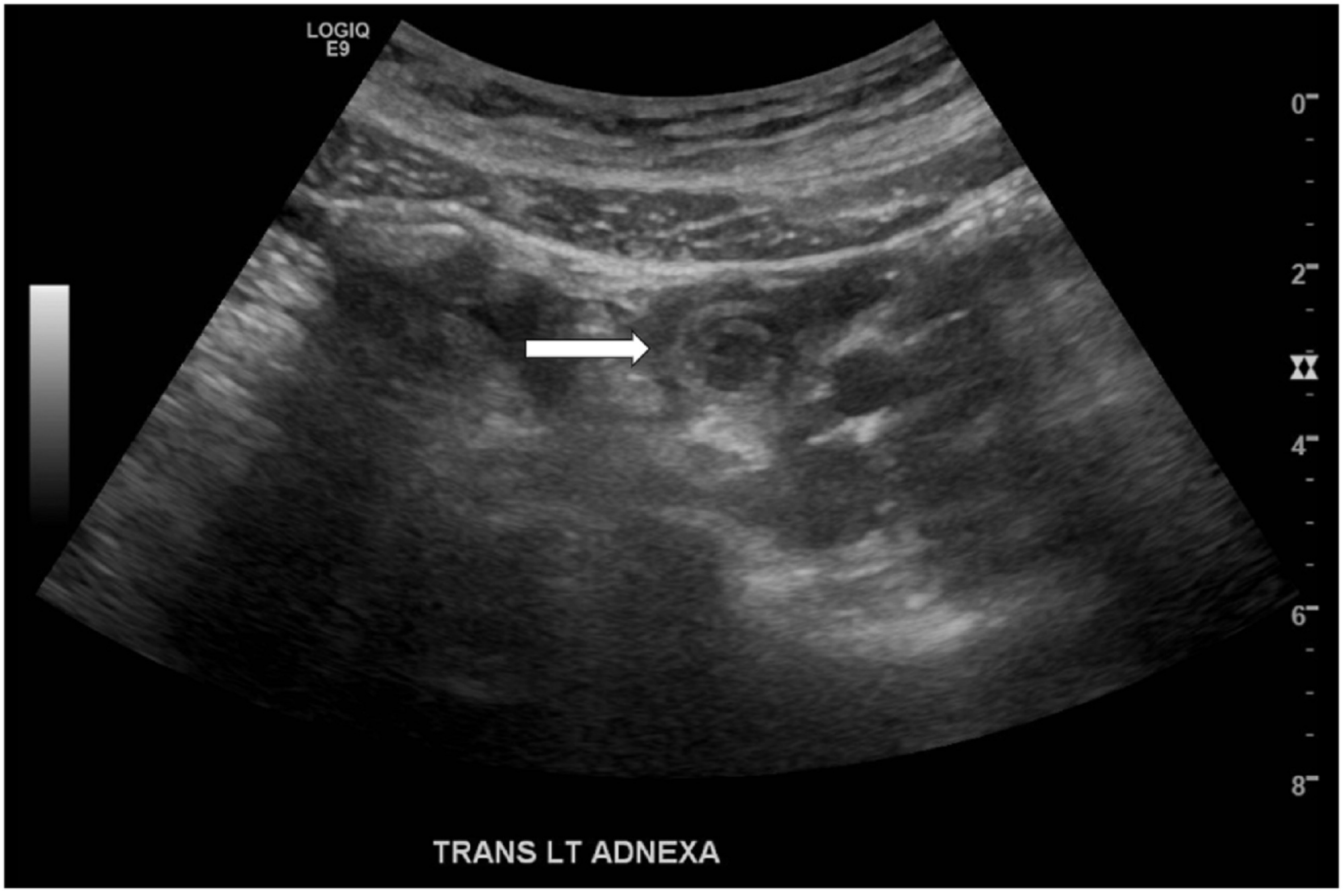
Transverse view of transabdominal pelvic ultrasound of the left adnexa revealing targetoid lesion of intussuscepted small bowel (white arrow).

A 22-year-old woman with a past medical history of pelvic inflammatory disease (PID) presented to the ED with a five-day duration of crampy, left lower quadrant abdominal pain associated with nausea and three episodes of nonbloody diarrhea. On review of systems, the patient endorsed new vaginal discharge over these five days. On arrival to the ED, the patient’s vital signs were a blood pressure of 128/82 mmHg, heart rate of 96 beats per minute, respiratory rate of 16 breaths per minute, pulse oximetry of 100% on room air, and oral temperature of 98.5⁰ Fahrenheit with reported pain level of seven on a scale of one to ten. Her physical exam was pertinent for mild tenderness to palpation of the abdomen in her left lower quadrant but without guarding, rigidity, or rebound tenderness. A pelvic exam revealed mild left adnexal tenderness and mild cervical motion tenderness. Laboratory studies to include a complete blood count, complete metabolic panel, lipase, qualitative urine pregnancy, urinalysis, potassium hydroxide smear, wet smear, and gonorrhea and chlamydia tests were completed, all with unactionable results. A pelvic ultrasound study with transabdominal and transvaginal approaches was ordered for further investigation. The transabdominal images of the left adnexa revealed a small bowel-small bowel intussusception, which resolved upon study completion (Figure 1). The patient returned from her ultrasound study reporting resolution of her pain and nausea. A repeat physical exam demonstrated interval resolution of her left lower quadrant tenderness. The patient was subsequently discharged with strict ED return precautions and instructions to follow up with her primary care physician. She did not return to the ED within the next seven days.

## Discussion

Transient small bowel intussusception has rarely been described in the literature [3-6]. Risk factors for transient small bowel intussusception include Crohn’s and celiac disease. However, many patients develop the condition without any known risk factors [3-6]. Though not a known risk factor for intussusception, active and resolved PID have both been associated with development of small bowel obstruction, thought to be caused by translocation of the infection and inflammatory process to the bowel wall and adhesion development, respectively [7-8]. And, both inflammatory processes and adhesions are well recognized sources of small bowel intussusception [2]. However, given our patient's PID was appropriately treated several months prior with negative ED pelvic microbiological testing, active infection and inflammation are less likely. Additionally, a small bowel intussusception due to adhesions would have a low likelihood of spontaneous resolution. Although idiopathic, transient small bowel intussusception has been captured incidentally on imaging performed in asymptomatic patients, the majority of symptomatic patients presented with nonspecific abdominal pain and without identification of a lead point [3-6, 9]. However, no publication to date has recorded resolution of symptoms with real-time resolution of the intussuscepted bowel diagnosed on pelvic ultrasound.

In the majority of cases referenced above, patients were diagnosed with CT of the abdomen, with reported accuracy ranging from 58% to 100% for diagnosing intussusception [2]. Abdominal ultrasound, the primary radiographic study for diagnosing intussusception in children, has been utilized to a lesser degree in adult diagnosis, likely due to reported accuracy estimates of approximately 50% [10]. This estimate is likely reflective of baseline operator skill, patient obesity, and gas-filled loops of distended bowel which may obscure diagnosis [2]. In this case, the pelvic ultrasound was obtained in our adult female patient due to initial suspicion for gynecologic pathology, where the transabdominal approach revealed the small bowel-small bowel intussusception and subsequent resolution. Unfortunately, the segments of intussuscepted small bowel were not specified, likely due to gynecologic focus of exam and prompt intussusception resolution. 

Transient small bowel-small bowel intussusception may resolve spontaneously. The correlation with documentation of intussusception resolution coinciding temporally with resolution of symptoms after radiographic study completion in the ED has not been previously described in the literature. One case, reported resolution of patient symptoms shortly after radiocontrast small bowel follow through performed the day following symptom onset [4]. Though not an ideal radiographic study, no lead point was identified on the pelvic ultrasound, and with symptom resolution, no further workup was pursued. Surgical exploration is an option utilized for refractory symptoms; bowel reduction due to persistent obstruction; and those at high risk for malignancy. However, there are no formal guidelines for surgical exploration for idiopathic, transient small bowel intussusception. Moreover, while larger small bowel intussusceptions are more likely to require surgical intervention, those measuring less than 3.5 cm in length are more likely self-limiting [11]. As such, in a low risk patient population, authors advocate for nonoperative treatment with symptom management and close follow up [4, 6].

## Conclusions

Intussusception is a rare but potentially serious etiology of abdominal pain in adults. Idiopathic, transient small bowel-small bowel intussusception is a scarcely reported phenomenon, usually diagnosed on CT scan of the abdomen. Here, we described a case of a nonpregnant, 22-year-old female with left lower quadrant pain, diagnosed with idiopathic, transient small bowel-small bowel intussusception on transabdominal pelvic ultrasound. Intussusception resolution occurred during the study with subsequent symptom and physical exam finding resolution, and the patient was uneventfully discharged without subsequent return to the ED.
